# Fighting *Staphylococcus epidermidis* Biofilm-Associated Infections: Can Iron Be the Key to Success?

**DOI:** 10.3389/fcimb.2021.798563

**Published:** 2021-11-30

**Authors:** Fernando Oliveira, Holger Rohde, Manuel Vilanova, Nuno Cerca

**Affiliations:** ^1^ Centre of Biological Engineering, Laboratory of Research in Biofilms Rosário Oliveira (LIBRO), University of Minho, Braga, Portugal; ^2^ Institut für Medizinische Mikrobiologie, Virologie und Hygiene, Universitätsklinikum Hamburg-Eppendorf, Hamburg, Germany; ^3^ Instituto de Investigação e Inovação em Saúde (i3S), Universidade do Porto, Porto, Portugal; ^4^ Instituto de Biologia Molecular e Celular (IBMC), Universidade do Porto, Porto, Portugal; ^5^ Instituto de Ciências Biomédicas de Abel Salazar, Universidade do Porto (ICBAS-UP), Porto, Portugal

**Keywords:** iron acquisition systems, regulation of iron acquisition, Siderophores, *S. epidermidis* biofilms, role of iron in infection

## Abstract

*Staphylococcus epidermidis* is one of the most important commensal microorganisms of human skin and mucosae. However, this bacterial species is also the cause of severe infections in immunocompromised patients, specially associated with the utilization of indwelling medical devices, that often serve as a scaffold for biofilm formation. *S. epidermidis* strains are often multidrug resistant and its association with biofilm formation makes these infections hard to treat. Their remarkable ability to form biofilms is widely regarded as its major pathogenic determinant. Although a significant amount of knowledge on its biofilm formation mechanisms has been achieved, we still do not understand how the species survives when exposed to the host harsh environment during invasion. A previous RNA-seq study highlighted that iron-metabolism associated genes were the most up-regulated bacterial genes upon contact with human blood, which suggested that iron acquisition plays an important role in *S. epidermidis* biofilm development and escape from the host innate immune system. In this perspective article, we review the available literature on the role of iron metabolism on *S. epidermidis* pathogenesis and propose that exploiting its dependence on iron could be pursued as a viable therapeutic alternative.

## Introduction

Health care-associated infections (HAIs) are a significant cause of morbidity and mortality worldwide and represent an increasing problem in modern medicine (Haque et al., 2018). More than 4 million patients are affected by HAIs every year in Europe, with an average prevalence rate of 7.1%, which accounts for an annual cost of approximately 7 billion (Allegranzi et al., 2011; European Centre for Disease Prevention and Control, 2018). In developing countries, the estimated prevalence rates of HAIs are even higher, ranging from 5.7% to 19.1% (pooled prevalence rate of 10.1%) (Pittet et al., 2008; Allegranzi et al., 2011). Patients admitted to intensive care units are particularly susceptible to these infections, not only due to their immunocompromised status, but also due to the extensive use of invasive procedures and devices (e.g., mechanical ventilators and catheters) (Vincent, 2003). Bloodstream infections, surgical site infections and urinary tract infections account for the majority of HAIs (Magill et al., 2014). According to the type of infection, the causative agents may vary, although staphylococci, and in particular *S. epidermidis*, play an important role in HAIs episodes (Zarb et al., 2012). The establishment of *S. epidermidis* as a successful nosocomial pathogen stems from its intrinsic ability to form biofilms on the surface of implantable medical devices, which is often accompanied by multidrug resistance (Águila-Arcos et al., 2017) and increased antibiotic tolerance (Dengler Haunreiter et al., 2019), rendering the treatment of HAIs extremely challenging. Therefore, a deeper understanding of their pathogenic mechanisms, particularly those associated with biofilm formation, is, more than ever before, pivotal in the identification of new antibacterial drug targets.

## Bacterial Biofilms

Our perception of how bacterial growth takes place in the environment, particularly during infection, has changed over the years. If on one hand bacteria were initially thought to grow mostly under a planktonic mode of growth, as it occurs artificially in a liquid culture, it is currently well-established that forming a biofilm is the preferred mode of growth for most bacterial species (Flemming et al., 2016). The classic definition of a biofilm is a structured community of microorganisms adhered to each other and/or to a surface, which is often embedded in a self-produced matrix of extracellular polymeric substance (Donlan and Costerton, 2002). Despite its frequent association with infectious diseases, this mode of growth is also adopted by non-pathogenic bacteria in different locations of the human body, such as skin (Brandwein et al., 2016) or gastrointestinal tract (De Vos, 2015). Therefore, the ability to grow as a biofilm can be generally regarded as a way bacteria employ to cope with harsh environments (Flemming et al., 2016). In *S. epidermidis* biofilm assembly follows a basic stepwise process comprising distinct stages: primary attachment of cells to a surface, accumulation of cells in multiple layers and maturation of the biofilm structure, and detachment of cells from the biofilm and their dispersal (Fey and Olson, 2010).

### *S. epidermidis* Biofilms in Infection

During infection, *S. epidermidis* face severe restriction in the availability of essential nutrients, a phenomenon usually referred to as “nutritional immunity” (Cassat and Skaar, 2013). Iron is one of those essential nutrients, which bacteria must acquire for their own cellular functions (Andrews et al., 2003). As a result, iron acquisition has been considered as a key process in bacterial pathogenicity (Skaar, 2010; Oliveira et al., 2021a). Interestingly, there has been increasing evidence suggesting that iron may also exert a modulatory effect over *S. epidermidis* biofilm formation (Oliveira et al., 2017), although the exact mechanisms are not completely understood. Nevertheless, the study of biofilms under iron-restricted conditions represents not only a closer approximation to the environmental conditions found in the human host, but it is also pivotal for a better comprehension of the molecular mechanisms behind biofilm formation in an infection scenario.

The matrix of *S. epidermidis* biofilms is a complex mixture of polysaccharides, proteins and nucleic acids. The polysaccharide intercellular adhesin (also known as poly-N-acetylglucosamine; PIA/PNAG), which biosynthesis is mediated by the products of the *ica* (intercellular adhesion) operon (Heilmann et al., 1996) was one of the first molecules found to be implicated in *S. epidermidis* biofilm accumulation (Ziebuhr et al., 1997). Since then, other factors mediating intercellular adhesion and biofilm accumulation have been identified such as the accumulation-associated protein (Aap) (Rohde et al., 2005), the extracellular matrix-binding protein (Embp) (Christner et al., 2010), or the small basic protein (Sbp) (Decker et al., 2015). The matrix of *S. epidermidis* biofilms has been shown to impede the penetration of antimicrobial molecules, phagocytic cells, reactive oxygen species, among others (Otto, 2012), which partly explains why *S. epidermidis* biofilm-associated infections are hard to eradicate or frequently relapse. Nevertheless, the understanding that the biofilm matrix acts solely as a physical barrier has been challenged over the years. In a study addressing biofilms formed by *S. epidermidis*, *Staphylococcus aureus*, *Escherichia coli*, and *Klebsiella pneumoniae* it was demonstrated that the ability of different classes of antibiotics to kill biofilm cells are independent of penetration (Singh et al., 2016). Another important issue about biofilm-associated infections is that biofilm cells employ different mechanisms to evade the host immune response. It was previously demonstrated that the diffusion of antibodies through *S. epidermidis* biofilms is not hindered by the biofilm matrix itself, but instead antibodies penetrate the matrix and bind to specific receptors within the matrix (i.e. PIA/PNAG), which reduce the available antibodies during opsonophagocytosis (Cerca et al., 2006). Inactivation of antimicrobial peptides (AMPs) and complement proteins has also been observed in *S. epidermidis* biofilms (Kristian et al., 2008). Moreover, biofilm-forming *S. epidermidis* strains were found to impair macrophage cell activation (Schommer et al., 2011). Another issue related with biofilms is the fact that cells adopting this mode of growth exhibit a decreased metabolic rate, which leads to lower efficiency of antibiotics whose action is dependent on actively growing cells (Cerca et al., 2005). Moreover, low pro-inflammatory properties have been attributed to dormant *S. epidermidis* biofilm cells (Cerca et al., 2011).

## Iron and Its Biological Importance

Considering that a large proportion of nosocomial infections is associated with biofilm formation and that *S. epidermidis* relies on iron acquisition to survive in the host environment, it is noteworthy to explore the link between these two bacterial processes. Iron belongs to the subfamily of transition elements and is one of the most abundant metals on Earth (Frey and Reed, 2012). It is a key nutrient for almost all living organisms, including bacteria, with very few exceptions (Archibald, 1983; Troxell et al., 2012), since it participates in essential biochemical processes, such as electron transfer and catalysis (Hudson et al., 2005). In nature, most iron exists under the form of two oxidative states: ferrous (Fe^2+^) and ferric (Fe^3+^) iron. Under aqueous, aerobic environments, Fe^2+^ is spontaneously oxidized to Fe^3+^, leading to the formation of ferric hydroxide (Morgan and Lahav, 2007). Additionally, the solubility of ferric hydroxide under neutral pH conditions usually found in the human body is extremely low (Schwertmann, 1991). To overcome this low solubility issue, superior organisms produce proteins (e.g. transferrin and ferritin) that are able to bind Fe^3+^ and maintain it stable while making it simultaneously available for biochemical processes (Brock, 1989).

Of note, the adult human body contains approximately 3-5 g of iron (Zhang and Enns, 2009). Even though this represents a large quantity, the levels of free ferric ion available in the body are kept to a minimum (~10^-24^ M) (Raymond et al., 2003; Waldvogel-Abramowski et al., 2014). Therefore, most of iron is complexed as Fe^2+^ in several proteins, such as metalloproteins. In these proteins, iron is mostly found in the form of heme prosthetic groups (Liu et al., 2014). Hemoglobin, a well-known metalloprotein present in erythroid precursors and mature erythrocytes, represents the major iron reservoir in humans (∼65%). The remaining iron is stored in hepatocytes, bound to ferritin, and within macrophages (Meynard et al., 2014). A small proportion can be found in muscles within myoglobin, or as part of other cellular iron-containing proteins (Soares and Hamza, 2016). Another fraction of the iron is present in the so-called labile iron pool, which consists of redox-active iron ions (both Fe^2+^ and Fe^3+^) bound to a variety of low affinity ligands (Kakhlon and Cabantchik, 2002).

### Bacterial Iron Acquisition Systems

During infection, *S. epidermidis* faces very harsh conditions, particularly iron restriction, once it reaches the bloodstream (Cassat and Skaar, 2013). The tiny amount of iron residing extracellularly is mostly bound by high affinity iron-binding proteins. This ensures that the concentration of free iron in body fluids and tissues can be as low as 10^-24^ M (Raymond et al., 2003). This is an extremely low level to support bacterial proliferation, as microorganisms typically require iron concentrations of approximately 10^-6^ M for growth (Miethke, 2013). Surprisingly, there is a lack of comprehensive studies available to date on the iron acquisition mechanisms in *S. epidermidis*, despite being a major source of bloodstream infections (Kleinschmidt et al., 2015). With very few exceptions (Archibald, 1983; Troxell et al., 2012), most pathogens rely on their ability to scavenge several biologically essential metals, including iron, for their survival, both *in vitro* and *in vivo* (Takase et al., 2000; Raymond et al., 2003; Dale et al., 2004; Beasley et al., 2009). ATP-binding cassette (ABC) transporters are among the most common bacterial iron acquisition systems (Cartron et al., 2006), which allow the uptake of iron bound either to host-derived proteins (e.g., transferrin) (Taylor and Heinrichs, 2002) or bacterial-derived siderophores (Beasley et al., 2009; Brillet et al., 2012) and hemophores (Rossi et al., 2001).

Siderophore-mediated iron uptake is a widely spread strategy among bacteria to survive in iron-restricted environments. While no siderophore has been described in *S. epidermidis* so far, there are findings suggesting that this species is able to produce at least one siderophore (Oliveira et al., 2017; Oliveira et al., 2021b). Siderophores are a class of small (usually less than 1 kDa), potent iron-chelating organic molecules with high affinity for Fe^3+^ (Wilson et al., 2016). Siderophores generally form hexadentate, octahedral, complexes with ferric ions in a 1:1 ratio of siderophore to iron (Hider and Kong, 2010). These molecules are synthesized intracellularly and secreted into the environment as iron-free compounds (Hider and Kong, 2010). Once Fe^3+^-siderophore complexes are formed, their transport across the bacterial cell membrane to the cytoplasm takes place through a myriad of uptake systems, particularly ABC transporters (Chu et al., 2010). The subsequent release of iron from high affinity siderophores may follow two different mechanisms: (i) enzymatic reduction of siderophore-bound Fe^3+^ to Fe^2+^ (Matzanke et al., 2004; Ganne et al., 2017) or (ii) enzyme-catalyzed siderophore hydrolysis (Lin et al., 2005). By the time bacterial iron requirements are met, the transcription of genes encoding iron transport systems is downregulated through the action of a repressor protein called Fur (Ferric uptake regulator) (Troxell and Hassan, 2013).

### Regulation of Iron Acquisition Systems

The expression of different bacterial virulence factors, particularly those associated with iron acquisition, is triggered by a decreased intracellular iron content (Porcheron and Dozois, 2015). The regulatory protein Fur is key in a conserved mechanism across bacteria responsible for the regulation of transcriptional responses to iron deprivation, and is now recognized as the canonical global iron-responsive regulator in bacteria (Troxell and Hassan, 2013). In general, when the intracellular iron content surpasses the level required for proper cellular function, there is an association of one Fe^2+^ ion with two Fur monomers. In its dimeric form, Fur is able to bind a conserved 19-bp DNA motif within the operator region of target genes, designated as Fur box, which blocks RNA polymerase and ultimately leads to repression of gene transcription (Fillat, 2014). Once the intracellular iron levels become depleted, the Fe^2+^ ion dissociates from the Fur dimer, the Fur box becomes unoccupied, and transcription of target genes is resumed (Fillat, 2014). Current knowledge about the involvement of iron and Fur as transcription regulators in *S. epidermidis* is limited but in *S. aureus*, Fur has been shown to regulate the transcription of iron acquisition (Hazmanian et al., 2003; Torres et al., 2006; Beasley et al., 2009) and other virulence-related genes (Torres et al., 2010), and was implicated in biofilm formation (Johnson et al., 2005).

## Interplay between iron and biofilm formation in *S. epidermidis*

As part of the normal microflora of human skin and mucosae (Grice et al., 2009), *S. epidermidis*, and to a lesser extent *S. aureus*, are frequent sources of biofilm infections associated with the use of indwelling medical devices (e.g., catheter systems, prosthetic joints, and a range of other polymer and metal implants) (McCann et al., 2008; Hogan et al., 2015; Lourtet-Hascoët et al., 2016) and are also implicated in more serious medical conditions (e.g., sepsis) (Kleinschmidt et al., 2015; Tong et al., 2015). Staphylococcal biofilms have been the focus of intensive research, and the role of iron in this process has been explored. However, the regulatory role of iron in *S. epidermidis* biofilms is not well understood, mostly due to the considerable lack of studies. The first study dates back to early 90s (Deighton and Borland, 1993) and demonstrated that most strains displayed an enhanced biofilm formation ability under conditions of iron limitation, although this phenotype was strain-dependent and, in some cases, only becomes apparent after a prolonged incubation period (48 hours). During the first 24 hours, this stimulatory effect was only evident for strains classified as weak or moderate biofilm producers, while iron limitation produced an inhibitory effect for strong biofilm producers. A renewed interest in this field was raised by the astonishing finding that catecholamine inotropic drugs, which are frequently administered in intensive-care units, significantly promote biofilm formation by *S. epidermidis* through sequestration of iron from transferrin (Neal et al., 2001; Lyte et al., 2003). The importance of iron for *S. epidermidis* biofilms is underscored by the fact that biofilm cells upregulate the transcription of genes involved in iron acquisition upon contact with human blood (França et al., 2014). In another study, it was demonstrated that the ability of different strains, including the reference strain *S. epidermidis* ATCC 35984 (RP62A), to form biofilms is strongly inhibited under iron-limiting conditions due to a delayed growth rate, reduced cell viability and impaired PIA/PNAG production (Oliveira et al., 2017). Iron excess (1 mM) produces a mildly detrimental effect in biofilm formation that is not related with PIA/PNAG (Oliveira et al., 2017). Siderophore biosynthesis or iron/siderophore transport systems, although not fundamental for planktonic growth under iron starvation, are absolutely required for biofilm formation under these conditions (Oliveira et al., 2021b).

## Siderophores and their role in bacterial pathogenesis

**Figure 1:**
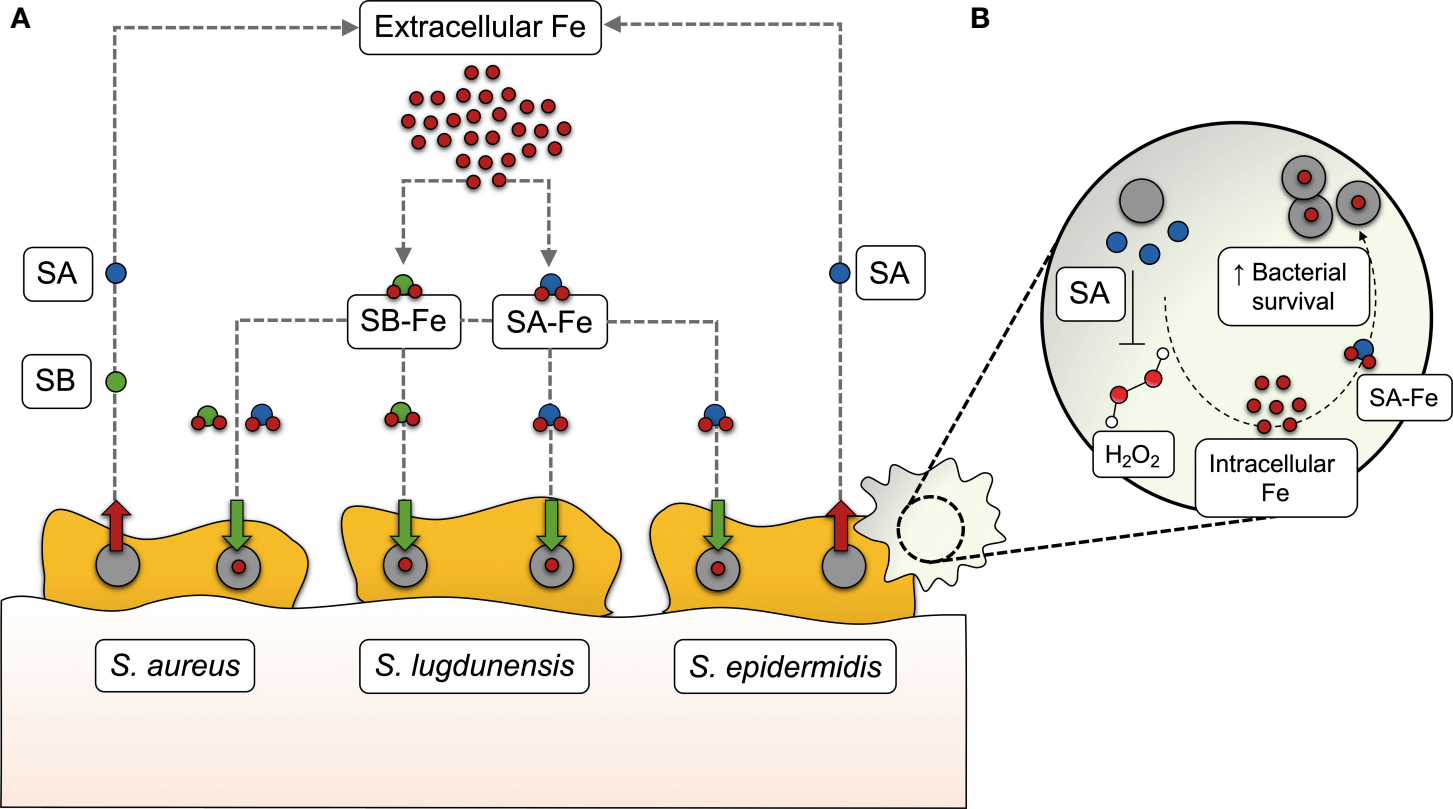
Siderophore-mediated iron acquisition in staphylococci. **(A)** While *S. aureus* produces two different siderophores (staphyloferrin A, SA, and staphyloferrin B, SB), *S. epidermidis* synthesizes SA only, and *S. lugdunensis* hijacks SA and SB from these two staphylococci species instead of producing them. After being secreted into the extracellular medium, SA and SB bind to extracellular iron (Fe) and make their way back to the cell as siderophore-iron complexes (SA-Fe and SB-Fe), providing the iron levels required for several bacterial processes, including biofilm formation. **(B)** In *S. epidermidis*, SA-mediated iron acquisition has recently been found to contribute to bacterial survival in human macrophages, by withstanding the action of certain reactive oxygen species, such as hydrogen peroxide (H_2_O_2_).

The perception of bacterial iron acquisition systems, particularly siderophores, as virulence factors is derived from the observation that their inactivation results in a measurable loss of virulence (Meyer et al., 1996; Rabsch et al., 2003; Montañez et al., 2005; Beasley et al., 2011; Reddy et al., 2013; Runci et al., 2019). One of the host mechanisms to counteract bacterial iron acquisition is the secretion of lipocalin 2 (Lcn2) by cells of the innate immune system, blocking the action of some siderophores, such as enterobactin (Flo et al., 2004). Bacteria overcome this issue through the production of diverse, functionally redundant siderophores, some of them being resistant to the action of Lcn2 (also referred to as “stealth siderophores”) (Fischbach et al., 2006; Bachman et al., 2011). This, together with the implication of siderophores in roles other than iron sequestration, such as the modulation of host cellular pathways (Holden and Bachman, 2015), seems to provide an adaptive advantage over the host immune response and contributes to bacterial pathogenesis. Assigning this kind of roles across different siderophores is difficult not only due to their structural and chemical diversity, but also because their biosynthesis may follow different pathways even in the most closely related species. Staphylococci are a paradigmatic example: *S. aureus* produces two different siderophores (staphyloferrins A and B) (Beasley et al., 2009; Cotton et al., 2009), *S. epidermidis* synthesizes staphyloferrin A only (Oliveira et al., 2021b), and *S. lugdunensis* hijacks siderophores from other staphylococci instead of producing them (Brozyna et al., 2014) (**Figure 1A**). Another striking difference regarding iron acquisition among staphylococci is that *S. aureus* and *S. lugdunensis* have a system dedicated to heme-bound iron acquisition, called iron-regulated surface determinant (Isd) (Torres et al., 2006; Heilbronner et al., 2011), which is absent in *S. epidermidis*. Therefore, the absence of the Isd system, in combination with the production of a single siderophore, underscores the relevance of siderophore-mediated iron acquisition in *S. epidermidis.*

The contribution of staphyloferrins in staphylococcal pathogenicity has been evidenced by the reduced virulence of siderophore-deficient *S. aureus* in different murine infection models (Dale et al., 2004; Nakaminami et al., 2017). This may be partly explained by the protective effect of staphyloferrin A to cells residing in host phagocytic cells. Deletion of *S. epidermidis sfaABCD*, which mediates siderophore biosynthesis, leads to compromised fitness in the macrophage intracellular milieu and increased susceptibility to reactive oxygen species (Oliveira et al., 2021b) (**Figure 1B**).

## Exploiting the bacterial dependence on iron for therapeutic purposes

The importance of iron and siderophores for biofilm formation and virulence across different nosocomial pathogens offers an opportunity for the development of new strategies to tackle biofilm-associated infections. Bacterial iron uptake systems, particularly siderophore and heme transport systems, can be exploited as gateways for the delivery of so-called “trojan horse” compounds into the bacterial cytoplasm. One possible approach is to use the heme synthetic analog gallium-protoporphyrin IX (GaPP) to facilitate the delivery of gallium into biofilms. GaPP has demonstrated remarkable antibiofilm activity against *S. aureus*, which is enhanced in combination with other compounds, such as gallium nitrate, iron chelating agents and conventional antibiotics (Chang et al., 2016; Richter et al., 2016; Richter et al., 2017a; Richter et al., 2017b; Choi et al., 2019a; Choi et al., 2019b). The use of GaPP is encouraged by the safety it demonstrated across cytotoxicity studies in different human cell lines, primary human cells and mice (Chang et al., 2016), although further studies are required on this matter. Nevertheless, care must be taken when selecting carrier molecules for the bacterial uptake of gallium, which should be based on the target species. For instance, despite the efficacy of GaPP against staphylococci, complexation of gallium with their native siderophore staphyloferrin A results in poor antimicrobial activity, raising some concerns about the suitability of native siderophores as carriers of gallium-based compounds (Kelson et al., 2013). The “trojan horse” strategy may also be employed for improved delivery of conventional antibiotics to pathogens through their conjugation with siderophores. Surprisingly, while there is evidence about the promising antibacterial activity of this group of compounds against Gram-positive and Gram-negative pathogens (Ji et al., 2012; Wencewicz et al., 2013; Ito et al., 2016; Ito-Horiyama et al., 2016), its efficacy against biofilms, in which antibiotic penetration is particularly dampened, remains unknown and is worth further investigation.

Iron acquisition-related molecules are also regarded as suitable target candidates for vaccine development since they display a good degree of conservation and their expression is readily induced as soon as pathogens invade the host and face nutritional immunity (Sheldon and Heinrichs, 2012). The Syntiron/Sanofi Pasteur consortium recently started preclinical trials on a multivalent vaccine based on four iron-regulated lipoproteins for the prevention of *S. aureus* skin and soft tissue infection (Syntiron, Sanofi Pasteur, 2018), although no detailed information has been made publicly available.

## Conclusions and Perspectives

*S. epidermidis* biofilm-associated infections are an increasing issue worldwide and have posed huge challenges to healthcare professionals. In the years to come, the global spread of multidrug-resistant lineages (Lee et al., 2018) may render the treatment of *S. epidermidis* biofilm-associated infections extremely difficult. Given this likely scenario, there is an urgent need to identify alternative bacterial targets for the development of novel anti-infective strategies. During the last years, we have been witnessing a renewed interest in bacterial iron acquisition mechanisms, mostly due to very promising findings underscoring the complex regulatory role of iron in biofilm formation, as well as the major role of siderophores in the virulence of several pathogens, including *S. epidermidis*. Consequently, this has put a spotlight on iron acquisition-related processes and brought a new hope for the development of a much-needed new generation of therapeutic strategies against life-threatening nosocomial infections. Nevertheless, there is still a long road ahead, as we still need to achieve a deeper understanding of the different biological roles that siderophores may assume in the pathogenicity of biofilm-associated infections and, most importantly, of the whole range of consequences of inhibiting iron acquisition in bacteria.

## Data Availability Statement

The original contributions presented in the study are included in the article/supplementary material. Further inquiries can be directed to the corresponding author.

## Author Contributions

NC and FO drafted the first version of the manuscript. HR and MV reviewed and edited the first version of the manuscript. All authors contributed to the article and approved the submitted version.

## Funding

FO is supported by the Fundação para a Ciência e a Tecnologia research project with reference PTDC/BIA-MOL/29553/2017, under the scope of COMPETE2020 (POCI-01-0145-FEDER-029553).

## Conflict of Interest

The authors declare that the research was conducted in the absence of any commercial or financial relationships that could be construed as a potential conflict of interest.

## Publisher’s Note

All claims expressed in this article are solely those of the authors and do not necessarily represent those of their affiliated organizations, or those of the publisher, the editors and the reviewers. Any product that may be evaluated in this article, or claim that may be made by its manufacturer, is not guaranteed or endorsed by the publisher.
